# Induction Chemoradiotherapy Followed by Surgical Resection for Clinical T3 or T4 Locally Advanced Non–Small Cell Lung Cancer

**DOI:** 10.1245/s10434-012-2302-x

**Published:** 2012-03-07

**Authors:** Kazuhiko Shien, Shinichi Toyooka, Katsuyuki Kiura, Keitaro Matsuo, Junichi Soh, Masaomi Yamane, Takahiro Oto, Mitsuhiro Takemoto, Hiroshi Date, Shinichiro Miyoshi

**Affiliations:** 1 Department of Thoracic Surgery, Okayama University Hospital, Okayama, Japan; 2 Department of Respiratory Medicine, Okayama University Hospital, Okayama, Japan; 3 Division of Epidemiology and Prevention, Aichi Cancer Center Research Institute, Nagoya, Japan; 4 Department of Radiology, Okayama University Hospital, Okayama, Japan; 5 Department of Thoracic Surgery, Kyoto University Graduate School of Medicine, Kyoto, Japan

## Abstract

**Purpose:**

To examine the usefulness of trimodality therapy in patients with clinical T3 or T4 (cT3–4) locally advanced non–small cell lung cancer (LA-NSCLC).

**Methods:**

Between 1997 and 2009, a total of 76 LA-NSCLC patients with cT3–4 underwent surgery. Among them, 36 patients underwent induction chemoradiotherapy with docetaxel and cisplatin plus concurrent radiation followed by surgery (IC group). The other 40 patients initially underwent surgery (IS group). The outcomes of the IC and IS groups were then investigated. To minimize possible biases caused by confounding treatment indications, we performed a retrospective cohort analysis by applying a propensity score (PS). Patients were divided into three groups according to PS tertiles, and comparisons between the IC and IS groups were made by PS tertile-stratified Cox proportional hazard models.

**Results:**

For the entire cohort, which had a median follow-up duration of 48 months, the 3- and 5-year overall survival rates were 83.8 and 78.9%, respectively, in the IC group, versus 66.8 and 56.5%, respectively, in the IS group (*P* = 0.0092). After adjustments for potentially confounding variables, the IC group continued to have a significantly longer overall survival than the IS group (*P* = 0.0045). In addition, when the analysis was limited to 52 patients with cT3–4N0 or N1 disease, the IC group had a significantly longer overall survival than the IS group after adjustments for confounding variables (*P* = 0.019).

**Conclusions:**

Our study indicates that trimodality therapy is highly effective in patients with cT3–4 LA-NSCLC.

**Electronic supplementary material:**

The online version of this article (doi:10.1245/s10434-012-2302-x) contains supplementary material, which is available to authorized users.

Lung cancer continues to be a major cause of cancer mortality worldwide. Almost half of all patients develop inoperable stage IV disease with metastasis to distant sites, and a quarter of all patients are diagnosed after the cancer has already spread to regional lymph nodes or directly beyond the primary site, resulting in a stage II or III disease.^1^ For stage II or III non–small cell lung cancer (NSCLC), surgical resection remains an important part of treatment.^2^

The N and T statuses determine the degree of local disease extension. N2 or N3 (N2–3) disease and T3 or T4 (T3–4) disease are considered locally advanced (LA) diseases. An N2–3 status generally indicates a need for definitive chemoradiotherapy (CRT) or induction CRT followed by surgery, rather than initial surgery.^3^ On the other hand, initial surgery is generally the treatment of choice for N0 or N1 (N0–1) disease. Regarding the T factor, whereas T3 tumors are basically candidates for initial surgery, such treatment is not recommended for T4 tumors, in principle, because of the large risk of an incomplete resection, especially when the tumors have highly invaded important structures. However, complete resections may be possible in patients with limited invasion of some structures including the carina, left atrium, superior vena cava, or pulmonary artery.^2^ In addition, T4 staging tends to be clinically overstaged, rather than understaged.^4^ Considering these factors, clinical T4 (cT4) disease is not a contraindication for surgery.

In the literature, the 5-year survival rate of patients with T3 disease who were treated with initial surgery has been reported to be 18–37%.^5^^–^ 7 Thus, the clinical outcome remains unsatisfactory, especially in patients with incomplete resection. We have used induction chemotherapy or CRT followed by surgery for the treatment of N2–3 LA-NSCLC disease since 1995.^8^^,^ 9 Trimodality therapy for stage III NSCLC has yielded a 3-year survival rate of more than 60%, which seems to be superior to the outcome of reported T3 disease even though N2–3 diseases were included in our study cohort. These results strongly suggested the usefulness of trimodality therapy for LA-NSCLC including T3 disease. Considering these, whereas the use of induction CRT for LA-NSCLC remains controversial except in patients with superior sulcus tumor, we have applied trimodality therapy for less advanced diseases including T3N0M0 and T3N1M0 based on the physician’s discretion in individual cases.^10^

As evidence regarding trimodality therapy for clinical T3 or T4 (cT3–4) without N2–3 disease in randomized controlled trials remains scarce, second-best evidence from retrospective studies comparing two approaches should be considered.^2^ In the present retrospective study, we examined the usefulness of trimodality therapy for patients with cT3–4 LA-NSCLC, compared with patients who underwent initial surgery. One serious concern with this approach is that the results may be biased by confounding indications. To avoid this possibility, we conducted a retrospective cohort analysis by applying a propensity score (PS) analysis in addition to the usual cohort analysis.

## PATIENTS AND METHODS

### Patient Selection and Evaluation

The records of 76 consecutive patients with cT3–4 invasive NSCLC who underwent pulmonary resection with nodal dissection between March 1997 and October 2009 at Okayama University Hospital, Okayama, Japan, were reviewed. The patient characteristics are shown in Table 1. Patients whose cT status was determined by tumor size more than 7 cm or presence of satellite nodules were excluded from this study. Among cN2 patients, pathologic N2 was confirmed in 8 patients who underwent a mediastinoscopy. Among 76 patients, 36 patients received induction CRT before resection (IC group), and 40 patients underwent initial surgery without receiving induction CRT (IS group). All 8 patients who had confirmed pathologic mediastinal nodal metastasis were treated with induction CRT followed by surgery. The International Association of the Study of Lung Cancer tumor, node, metastasis system for NSCLC, 7th edition, was used for disease staging.^11^

**TABLE 1:** Patient characteristics (unadjusted)

Variable		IC (*n* = 36)	IS (*n* = 40)	*P*
Age, y, median (range)		58 (33–74)	68 (48–81)	0.0005
Sex, M/F		30/6	35/5	0.75
Histology, Sq/Ad/AdSq/LC		17/17/1/1	22/15/1/2	0.80
Performance status, 0/1		26/10	31/9	0.61
Period of treatment, 1997–2003/2004–2009		14/22	23/17	0.10
c stage	T3 N0	5	18	<0.001
IIB	T3 N1/N2	3/7	11/4	
IIIA	T4 N0/N1	3/5	5/2	
IIIB	T3 N3	4	0	
	T4 N2	9	0	
Involved structures				
cT3	Chest wall	12	28^a^	
	Parietal pleura	5	12	
	Rib or muscle	7	16	
	Diaphragm	0	3^a^	
	Pericardium	1	4^a^	
	Mediastinal pleura	3	1	
	<2 cm carina	3	1	
cT4	Atrium	1	0	
	Great vessel	13^a^	7	
	Esophagus	2^a^	0	
	Vertebral body	1^a^	0	
	Carina	2	0	
Superior sulcus		4	1	

*IC* induction chemoradiotherapy, *IS* initial surgery, *Sq* squamous cell carcinoma, *Ad* adenocarcinoma, *AdSq* adenosquamous carcinoma, *LC* large cell carcinoma

^a^ Multiple structures were involved: chest wall with diaphragm (*n* = 1), with pericardium (*n* = 2), diaphragm with pericardium (*n* = 1), great vessel with esophagus (*n* = 1), with vertebral body (*n* = 1)

Clinical stage was determined by chest radiography, bronchoscopy, computed tomography (CT) of the chest and upper abdomen, magnetic resonance imaging (MRI) of the brain, and a radionuclide bone scan or ^18^F-fluorodeoxyglucose positron emission tomography (PET)/CT scan. The institutional review board approved the study, and informed consent was obtained from all the patients.

### Induction CRT

The application of trimodality therapy was left to the physician’s discretion. Basically, induction CRT was administered to patients who had mediastinal nodal metastases confirmed by a mediastinoscopy or large and invasive tumors that would have made the achievement of a complete resection with a pathologic safety margin difficult. The criteria and treatment regimen for induction CRT have been previously described.^9^ In brief, docetaxel (40 mg/m2) was administered intravenously followed by cisplatin (40 mg/m^2^) before radiotherapy on days 1 and 8. The chemotherapy was repeated at a 3- or 4-week interval. Radiotherapy was started on the first day of chemotherapy and total radiation dose of 46 gray (Gy) was planned, in principle, using a conventional fractionation (2 Gy/day). Dose escalation of the radiotherapy was allowed for poorly responding tumors up to 60 Gy.

After induction CRT, the patients were restaged and those without progressive disease were scheduled to receive surgery within 6 weeks of the completion of the induction therapy. The radiologic response of lesions was classified into four categories as described in previous study: complete response, partial response, stable disease, and progressive disease.^9^^,^ 12 The pathologic response to induction CRT was classified into three groups: pathologic complete response, pathologic major response, and pathologic minor response.^13^

### Surgical Resection, Adjuvant Treatment, and Follow-up

The surgical procedure was determined on the basis of the extent of disease. Although a lobectomy was preferred, a bilobectomy, sleeve resection, or pneumonectomy was performed in cases requiring such procedures because of the location of the primary tumor or metastatic nodal invasion. All the patients underwent complete ipsilateral mediastinal and subcarinal nodal dissection. For cN3 disease (*n* = 4), the contralateral hilar (*n* = 1) or ipsilateral supraclavicular (*n* = 3) lymph nodes were dissected according to the initial disease extent. Resection with reconstruction of the chest wall or major vessels was performed, if necessary. In the IC group, the bronchial stump was covered with pericardial fat tissue or an intercostal muscle pedicle. When a sleeve resection was performed, the greater omentum was used to wrap the anastomosis. Postoperative treatment was left to the physician’s discretion. Follow-up protocol after surgery is as follows: chest and abdominal CT or PET/CT scan and enhanced brain MRI were repeated every 3 months for 2 years. From 3 to 5 years, these image analyses were repeated every 6 months. After 5 years, chest X-ray was repeated every year.

### Statistical Methods

We calculated the PS by logistic regression based on available factors that were considered to be potentially associated with patient selection and the pscore command in Stata, version 11 (StataCorp, College Station, TX). Seven such factors that were included in the PS calculation were age, sex, histology, performance status, clinical stage, operation type, year of treatment (Supplementary Material 1). After the PS calculation, the subjects were divided into 3 groups according to the PS tertile to sustain comparability between the IC and the IS group within each stratum.

In this study, overall survival (OS) was defined as the primary end point and disease-free survival (DFS) as the secondary end point. OS and DFS were calculated from the date of initial treatment until the date of death or the last follow-up for OS and until confirmed disease recurrence by cross-sectional imaging studies or death for DFS. We used the PS to control potential confounding by treatment indication for the induction CRT on survival. We compared end points for the IC and IS groups in three patterns: (1) comparison using all the patients in the cohort (*n* = 76), (2) comparison among cN0–1 patients (*n* = 52), and (3) comparison of a selected cohort in which IC patients were individually matched with IS patients by PS in all the patients (*n* = 32), and cN0–1 patients (*n* = 24). For pattern 1 and 2, the IC and IS groups were compared by a stratified logrank test and a stratified multivariate Cox proportional hazard model with PS tertile as a stratification factor. For pattern 3, the IC and IS groups were compared by usual logrank test and multivariate Cox proportional hazard model.

The baseline characteristics of the IC and IS groups were compared by the Wilcoxon rank sum test for continuous variables and the Fisher’s exact test for categorical variables, as appropriate. Data were analyzed by Stata, version 11. *P* values of less than 0.05 were considered significant for comparison of characteristics. We applied *P* value less than 0.01 as significant for survival analysis to avoid inflation of alpha error by multiple comparisons.

## RESULTS

### Patient Characteristics

Between March 1997 and October 2009, a total of 76 LA-NSCLC patients with cT3–4 disease underwent surgical resection. The patient characteristics stratified according to therapeutic modality are shown in Table 1. A difference in the patient backgrounds was noted for age and clinical stage. The median age was significantly younger and the clinical stage was significantly higher in the IC group than in the IS group (age, *P* = 0.0005; stage, *P* < 0.001).

### Toxicities and Response to Induction CRT

Out of the 36 patients in the IC group, 23 completed the planned CRT. The toxicities were similar to those described in our previous report.^9^ The chemotherapy dose was modified because of toxicity in 12 patients. The total radiation dose was 20 Gy in 1 patient, 40 Gy in 8 patients, 46 Gy in 19 patients, and more than 50 Gy in 8 patients. The clinical response to induction therapy was partial response in 25 patients, stable disease in 11 patients, and complete response or progressive disease in none. The pathologic response in the resected specimens was estimated. Nineteen patients (53%) exhibited a pathologic complete response, 17 patients (47%) exhibited a pathologic major response, and none of the patients exhibited a pathologic minor response.

### Surgical Resection, Postoperative Complications, and Adjuvant Treatment

The surgical procedures and postoperative complications are shown in Table 2. All 76 patients underwent surgical resection. In the IC group, all the patients underwent complete tumor resection. In the IS group, 37 (93%) of the 40 patients underwent complete tumor resection; two patients had pleural dissemination that was diagnosed pathologically after surgery, and one patient had a microscopically positive surgical margin.

**TABLE 2:** Surgical procedure and postoperative complications

Variable	IC (*n* = 36)	IS (*n* = 40)	*P*
Operation type			0.015
Lobectomy	21	34	
Sleeve lobectomy	7	1	
Bilobectomy	5	1	
Pneumonectomy	3	4	
Combined resection, yes/no	27/9	35/5	0.24
Chest wall	12^a^	25^a^	
Parietal pleura	8	10	
Rib or muscle	4	15	
Diaphragm	0	6^a^	
Pericardium	0	5^a^	
Mediastinal pleura	6	1	
Atrium	1	0	
Great vessel	7^a^	5^a^	
Recurrent nerve	1^a^	0	
Esophagus	1	0	
Carina	1	0	
Complete resection, yes/no	36/0	37/3	0.24
Postoperative complication,^b^ yes/no	18/18	15/25	0.36
Pneumonia	2	6	
Empyema	2	0	
Hemorrhage, reoperation	0	1	
Chylothorax	1	0	
Bronchopleural fistula	1	0	
Atrial arrhythmia	2	2	
Hoarseness	2	4	
Effusion	0	2	
Brachial plexus palsy	1	0	
Radiation pneumonitis	4	0	
Postoperative morality	0	1	

*IC* induction chemoradiotherapy, *IS* initial surgery

^a^ Multiple structures were resected; (Induction CRT group) chest wall with great vessel (*n* = 1), with recurrent nerve (*n* = 1), (Initial surgery group) chest wall with diaphragm (*n* = 1), with pericardium (*n* = 2), diaphragm with pericardium (*n* = 2), chest wall with diaphragm and great vessel (*n* = 1)

^b^ The number of each event was described

One patient in the IS group died after a left pneumonectomy because of acute respiratory failure. Pulmonary complications were the most common type of postoperative complication in both groups. No significant difference in the rate of postoperative complications was observed between the groups.

In the IC group, 14 patients underwent adjuvant chemotherapy. In the IS group, 14 patients underwent adjuvant chemotherapy, seven patients underwent adjuvant radiotherapy, three patients underwent adjuvant CRT.

### Survival and Relapse Pattern

At a median follow-up period of 48 months, five patients in the IC group and 13 patients in IS group had died of NSCLC. One patient in the IC group and three patients in the IS group had died of other causes. Seven patients in the IC group and 17 patients in the IS group had experienced a disease relapse. The disease recurrence patterns were classified as local sites (surgical margin, intrapulmonary, regional lymph node, and pleural cavity) and distant sites. The initial recurrent patterns were as follows: in the IC group, 1 patient (3%) developed a recurrence at local site, 3 patients (8%) developed recurrences at distant sites, and 3 patients (8%) developed recurrences at both sites; in the IS group, 8 patients (20%) developed recurrences at local sites, 5 patients (13%) developed recurrences at distant sites, and 4 patients (10%) developed recurrences at both sites.

**FIG. 1:**
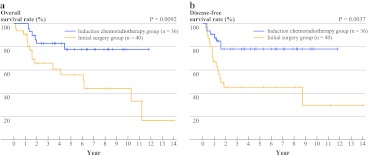
Kaplan-Meier curves showing the unadjusted **a** OS and **b** DFS rates for patients in the IC and IS groups

In analysis pattern 1 with the unadjusted model, the 3- and 5-year OS rates were 83.8 (95% confidential interval [CI] 65.3–92.9) and 78.9% (95% CI 58.2–90.1) in the IC group, versus 66.8 (95% CI 46.6–80.8) and 56.5% (95% CI 35.1–73.2) in the IS group (Fig. 1). The patients in the IC group showed significantly longer OS and DFS times than those in the IS group (OS, *P* = 0.0092; DFS, *P* = 0.0037). Subset analyses identified several factors that were related to a favorable outcome: the initial disease stage, sex, histology, and pathologic response. Only the use of induction therapy was significantly associated with the outcome. There was no significant difference in OS and DFS between those with or without adjuvant therapy in both the IC and IS groups.

The comparison of the characteristics between the IC and IS groups according to the PS tertile indicated an equivalent distribution of the background characteristics (Supplementary Material 2). When the PS was taken into consideration, the patients in the IC group continued to exhibit a significantly longer OS than those in the IS group according to a stratified logrank test (*P* = 0.0045). A multivariate analysis showed a significantly better outcome in the IC group (hazard ratio [HR], 0.068; 95% CI 0.013–0.35; *P* = 0.001) (Supplementary Material 3).

**FIG. 2:**
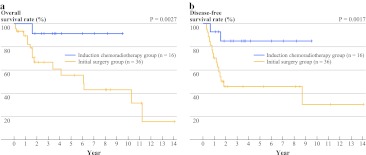
Kaplan-Meier curves showing the unadjusted **a** OS and **b** DFS rates for patients with cT3–4 and cN0–1 disease

The OS and DFS curves for 52 patients with clinical N0 or N1 (cN0–1) (analysis pattern 2) are shown in Fig. 2. The 3- and 5-year OS rates were both 92.3% (95% CI 56.6–98.9) in the IC group (*n* = 16) and were 67.7 (95% CI 46.6–81.9) and 56.4% (95% CI 33.9–73.8) in the IS group (*n* = 36), respectively. In this cohort, the OS and DFS times in the IC group were significantly longer than those in the IS group (OS, *P* = 0.027; DFS, *P* = 0.017). In addition, the PS values were calculated in this cohort, and survivals were analyzed after stratification according to the PS tertile (Supplementary Material 1). A comparison of the characteristics between the IC and IS groups according to the PS tertile is shown in Supplementary Material 4. When the PS was taken into the consideration, the patients in the IC group continued to exhibit a significantly longer OS than those in the IS group (a stratified logrank test*, P* = 0.019). A multivariate analysis continued to exhibit a significantly better outcome in the IC group in this cohort (HR, 0.068; 95% CI 0.00043–0.28; *P* = 0.006, Supplementary Material 5).

### PS Matching Analysis (Analysis Pattern 3)

Among all 76 patients, 32 patients were extracted from each group using PS matching. The patient characteristics were matched as shown in Table 3. After adjustments for potentially confounding variables, the patients in the IC group continued to exhibit a better outcome than those in the IS group (*P* = 0.00067). In addition, among 52 patients with cN0–1 disease, 24 patients were extracted using PS matching (Supplementary Material 6). After adjustments, the patients in the IC group also exhibited a better outcome those than the IS group (*P* = 0.026).

**TABLE 3:** Patient characteristics of PS matched cohort

Variable		IC (*n* = 16)	IS (*n* = 16)	*P*
Age, y, median (range)		64 (51–74)	64 (48–75)	0.82
Sex, M/F		13/3	13/3	1.00
Histology, Sq/Ad/AdSq		7/8/1	9/6/1	0.85
Performance status, 0/1		13/3	13/3	1.00
c stage, IIB/IIIA/IIIB		3/9/4	3/13/0	0.14
Involved structures				
cT3	Chest wall	7	9	
	Parietal pleura	3	5	
	Rib or muscle	4	4	
	Diaphragm	0	1^a^	
	Pericardium	0	2^a^	
	Mediastinal pleura	2	0	
	<2 cm carina	1	0	
cT4	Great vessel	6^a^	5	
	Esophagus	1^a^	0	
Superior sulcus		2	0	
Operation type				0.79
Lobectomy		13	12	
Sleeve lobectomy		1	0	
Bilobectomy		1	1	
pneumonectomy		1	3	
Period of treatment, y, 1997–2003/2004–2009		7/9	9/7	0.72
Combined resection, yes/no		14/2	15/1	1.00
Chest wall		6	9^b^	
Parietal pleura		2	6	
Rib or muscle		4	3	
Diaphragm		0	4^b^	
Pericardium		0	1^b^	
Mediastinal pleura		4	1^b^	
Great vessel		4	3	

*PS* propensity score, *IC* induction chemoradiotherapy, *IS* initial surgery, *Sq* squamous cell carcinoma, *Ad* adenocarcinoma, *AdSq* adenosquamous carcinoma

^a^ Multiple structures were involved: diaphragm with pericardium (*n* = 1), great vessel with esophagus (*n* = 1)

^b^ Multiple structures were resected; chest wall with diaphragm (*n* = 1), with diaphragm and great vessel (*n* = 1), diaphragm with pericardium (*n* = 1)

## DISCUSSION

We previously reported that induction CRT followed by surgery is a promising treatment for patients with stage III NSCLC.^9^ The present study focused on cT3–4 LA disease involving adjacent structures and compared the outcome of trimodality therapy with initial surgery in this population. Our study showed that trimodality therapy significantly prolonged the OS and DFS times. Although the present study was retrospective in design, a PS matching analysis reduced the biases inherent in this series.

A significantly high incidence of postoperative complications after induction treatments has been reported, particularly after a pneumonectomy.^14^ In the present study, no treatment-related mortalities were observed in the IC group. Although no significant difference in the rate of postoperative complication was observed between the groups, severe complications including bronchopleural fistula and empyema occurred in the IC group. This outcome suggests the importance of a meticulous surgical procedure and perioperative management.

The rationale for the use of induction treatments is to prevent cancer cell microresidues at local sites and to eradicate micrometastatic disease at distant sites.^15^ Indeed, the outcomes of the present study support this rationale. First, local recurrences occurred in 11% of the patients in the IC group and 30% in the IS group. The control of local recurrences using induction CRT is thought to contribute to a better likelihood of achieving a complete resection. Furthermore, nodal metastasis is known to be significantly more common among patients with visceral pleura invasion, possibly through the subpleural lymphatics to the hilar and mediastinal lymph nodes.^16^ The therapeutic effect of induction CRT on this afferent pathway may have contributed to the better outcome of the IC group. Second, distant metastases occurred in 17% of the patients in the IC group and 23% of the patients in the IS group. These frequencies are similar even though the IC group included more patients with an advanced disease stage, compared with the IS group. This outcome suggests the effectiveness of induction therapy for eradicating distant micrometastasis.

In this study, all 13 patients with c-IIIB disease (4 of T3N3M0 and 9 of T4N2M0) were treated with trimodal therapy, but mediastinal nodal metastasis was only confirmed in three patients. Recent guidelines do not recommend surgery for T4N2 or more advanced disease, nor do they recommend induction chemotherapy or CRT followed by surgery for N3 disease.^1^^,^ 3 ^,^ 17 Although the c-IIIB stage may have been overestimated in our series, all the c-IIIB patients were treated with trimodality therapy, and their clinical outcomes seemed to be acceptable (3-year survival rate of 75%).

Finally, our induction CRT regimen was thought to contribute to the favorable outcome of the trimodality therapy. Our regimen originated from the same combination of docetaxel and cisplatin with concurrent radiation that is used for unresectable LA-NSCLC.^18^ A recent study showed that concurrent CRT with docetaxel and cisplatin is associated with a favorable prognosis compared with mitomycin, vindesine, and cisplatin (MVP) in patients with unresectable LA-NSCLC.^19^ Of note, Stupp et al. reported the excellent prognosis (40% of 5-year OS rate) of stage IIIB patients treated with trimodal therapy with docetaxel and cisplatin.^20^

In conclusion, our results strongly suggest that this combined modality treatment is highly effective in patients with cT3–4 LA-NSCLC, compared with initial surgery.

## Electronic Supplementary Material

Below is the link to the electronic supplementary material.

Supplementary material 1 (DOC 95 kb)
