# Supplementary material for: Induction Chemoradiotherapy Followed by Surgical Resection for Clinical T3 or T4 Locally Advanced Non–Small Cell Lung Cancer
Source: Ann Surg Oncol. 2012 Mar 7;19(8):2685–92. doi: 10.1245/s10434-012-2302-x (PMC3404289; doi:10.1245/s10434-012-2302-x)
Supplement: Supplementary file 1 — Supplementary material 1 (DOC 95 kb) [file 10434_2012_2302_MOESM1_ESM.doc]

***Supplementary materials***

**Supplementary material 1.** Covariates that are considered to concern selection of the types of treatment

| Covariates | Category (All patients, n = 76) | Category (cT3-4/N0-1, n = 52) |
| --- | --- | --- |
| Age at diagnosis | Continuous value | Continuous value |
| Sex | Male vs. female | Male vs. female |
| Histology | Ad vs. Sq, AdSq, and LC | Ad vs. others |
| Performance status | 0 vs. 1 | 0 vs. 1 |
| c-Stage | IIB vs. IIIA/IIIB | IIB vs. IIIA |
| Operation type | Lobectomy vs. sleeve lobectomy, bilobectomy, and pneumonectomy | Lobectomy vs. sleeve lobectomy, bilobectomy, and pneumonectomy |
| Year of treatment | Continuous value | Continuous value |

Ad, adenocarcinoma; Sq, squamous cell carcinoma; AdSq, adenosquamous carcinoma; LC, large cell carcinoma

**Supplementary material 2.** Patient Characteristics stratified by PS tertile

| Variables | Tertile 1 | | | Tertile 2 | | | Tertile 3 | | |
| --- | --- | --- | --- | --- | --- | --- | --- | --- | --- |
|  | IC | IS | *P* | IC | IS | *P* | IC | IS | *P* |
| No. of patients | 3 | 23 |  | 11 | 14 |  | 22 | 3 |  |
| Median age (y) | 72 | 69 | 0.81 | 62 | 64 | 0.49 | 55 | 55 | 0.80 |
| Sex,  Male  Female | 2  1 | 21  2 | 0.32 | 9  2 | 12  2 | 1.00 | 19  3 | 2  1 | 0.42 |
| Histology,  Sq  Ad  AdSq  LC | 1  2  0  0 | 13  8  0  2 | 0.65 | 5  5  1  0 | 8  5  1  0 | 0.84 | 11  10  0  1 | 1  2  0  0 | 1.00 |
| Performance status,  0  1 | 2  1 | 17  6 | 1.00 | 10  1 | 11  3 | 0.60 | 14  8 | 3  0 | 0.53 |
| Period of treatment (y),  1997-2003  2004-2009 | 3  0 | 14  9 | 0.53 | 5  6 | 8  6 | 0.70 | 6  16 | 1  2 | 1.00 |
| c-Stage,  IIB  IIIA  IIIB | 1  1  1 | 14  9  0 | 0.16 | 3  4  4 | 4  10  0 | 0.046 | 1  13  8 | 0  3  0 | 0.58 |
| Operation type  Lobectomy  Sleeve lobectomy  Bilobectomy  Pneumonectomy | 2  0  0  1 | 22  0  0  1 | 0.22 | 10  0  1  0 | 10  1  0  3 | 0.23 | 9  7  4  2 | 2  0  1  0 | 0.67 |

PS, propensity score; IC, induction chemoradiotherapy; IS, initial surgery; Sq, squamous cell carcinoma; Ad, adenocarcinoma; AdSq, adenosquamous carcinoma; LC, large cell carcinoma

**Supplementary material 3.** Stratified multivariate analysis model for overall survival

| Factors | HR | *P* | 95% CI |
| --- | --- | --- | --- |
| Treatment, IC vs. IS | 0.068 | 0.001 | 0.013-0.35 |
| c-Stage, IIIB / IIIA / IIB | 2.13 | 0.13 | 0.80-5.65 |
| Sex, female vs. male | 0.68 | 0.60 | 0.17-2.81 |
| Performance status, 1 vs. 0 | 1.54 | 0.45 | 0.17-2.81 |
| Histology, Ad vs. others | 1.66 | 0.40 | 0.51-5.41 |
| Operation type, lobectomy vs. others | 0.37 | 0.13 | 0.10-1.33 |
| Period of treatment (y), 2004-2009 vs. 1997-2003 | 0.31 | 0.031 | 0.11-0.90 |

CI, confidential interval; IC, induction chemoradiotherapy; IS, initial surgery; Ad, adenocarcinoma

**Supplementary material 4.**  Patient Characteristics stratified by propensity score tertile (T3-4N0-1)

| Variables | Tertile 1 | | Tertile 2 | | | Tertile 3 | | |
| --- | --- | --- | --- | --- | --- | --- | --- | --- |
|  | IC | IS | IC | IS | *P* | IC | IS | *P* |
| No. of patients | 0 | 18 | 3 | 14 |  | 13 | 4 |  |
| Median age (y) |  | 69 | 69 | 67 | 1.00 | 55 | 55 | 1.00 |
| Sex,  Male  Female |  | 17  1 | 2  1 | 11  3 | 1.00 | 11  2 | 3  1 | 1.00 |
| Histology,  Sq  Ad  AdSq  LC |  | 13  3  1  1 | 1  2  0  0 | 5  8  0  1 | 1.00 | 5  8  0  0 | 1  3  0  0 | 1.00 |
| Performance status,  0  1 |  | 14  4 | 2  1 | 11  3 | 1.00 | 11  2 | 4  0 | 1.00 |
| Period of treatment (y),  1997-2003  2004-2009 |  | 11  7 | 2  1 | 8  6 | 1.00 | 1  12 | 1  3 | 0.43 |
| c-Stage,  IIB  IIIA |  | 10  8 | 1  2 | 7  7 | 1.00 | 4  9 | 1  3 | 1.00 |
| Operation type  Lobectomy  Sleeve lobectomy  Bilobectomy  Pneumonectomy |  | 16  0  0  2 | 3  0  0  0 | 11  1  0  2 | 1.00 | 6  3  3  1 | 3  0  1  0 | 0.82 |

IC, induction chemoradiotherapy; IS, initial surgery; Sq, squamous cell carcinoma; Ad, adenocarcinoma; AdSq, adenosquamous carcinoma; LC, large cell carcinoma

**Supplementary material 5.** Stratified multivariate analysis model for overall survival (T3-4N0-1)

| Factors | HR | *P* | 95% CI |
| --- | --- | --- | --- |
| Treatment, IC vs. IS | 0.011 | 0.006 | 0.00043-0.28 |
| c-Stage, IIIA vs. IIB | 1.31 | 0.70 | 0.33-5.29 |
| Sex, female vs. male | 1.37 | 0.77 | 0.16-11.7 |
| Performance status, 1 vs. 0 | 4.99 | 0.079 | 0.83-30.1 |
| Histology, Ad vs. others | 1.90 | 0.41 | 0.41-8.85 |
| Operation type, lobectomy vs. others | 0.082 | 0.016 | 0.011-0.64 |
| Period of treatment (y), 2004-2009 vs. 1997-2003 | 0.031 | 0.004 | 0.0029-0.34 |

HR, hazard ratio; CI, confidential interval; IC, induction chemoradiotherapy; IS, initial surgery; Ad, adenocarcinoma

**Supplementary material 6.** Patient characteristics of PS matched cohort (T3-4N0-1)

| Variables | | IC  (n = 12) | IS  (n = 12) | *P* |
| --- | --- | --- | --- | --- |
| Median age, range (y) | | 59 , 51-72 | 66 , 48-79 | 0.37 |
| Sex, Male / Female | | 9 / 3 | 10 / 2 | 1.00 |
| Histology, Sq / Ad / AdSq / LC | | 4 / 8 / 0 / 0 | 6 / 4 / 1 / 1 | 0.29 |
| Performance status, 0 / 1 | | 10 / 2 | 11 / 1 | 1.00 |
| c-Stage, IIB / IIIA | | 4 / 8 | 3 / 9 | 1.00 |
| Involved structures | |  |  |  |
| cT3  cT4 | Chest wall  Parietal pleura  Rib or muscle  Mediastinal pleura  < 2cm carina  Great vessel  Esophagus | 6  2  4  0  1  5*  1* | 6  3  3  1  0  5  0 |  |
| Superior sulcus | | 4 | 0 |  |
| Operation type  Lobectomy  Sleeve lobectomy  Bilobectomy  pneumonectomy | | 7  3  1  1 | 11  0  0  1 | 0.15 |
| Period of treatment (y), 1997-2003 / 2004-2009 | | 2 / 10 | 6 / 6 | 0.19 |
| Combined resection, Yes / No  Chest wall  　 Parietal pleura  Rib or muscle  Diaphragm  Mediastinal pleura  Great vessel | | 10 / 2  6  1  5  0  1  3 | 10 / 2  7**  2  5  1**  1  3** | 1.00 |

*Multiple structures were involved: great vessel with esophagus (n = 1), **Multiple structures were resected; chest wall with diaphragm and great vessel (n = 1). PS, propensity score; IC, induction chemoradiotherapy; IS, initial surgery; Sq, squamous cell carcinoma; Ad, adenocarcinoma; AdSq, adenosquamous carcinoma; LC, large cell carcinoma
